# Association of Sepsis-Related Mortality with Early Increase of TIMP-1/MMP-9 Ratio

**DOI:** 10.1371/journal.pone.0094318

**Published:** 2014-04-11

**Authors:** Leonardo Lorente, María M. Martín, Jordi Solé-Violán, José Blanquer, Lorenzo Labarta, César Díaz, Juan M. Borreguero-León, Josune Orbe, José A. Rodríguez, Alejandro Jiménez, José A. Páramo

**Affiliations:** 1 Intensive Care Unit, Hospital Universitario de Canarias, La Laguna, Santa Cruz de Tenerife, Spain; 2 Intensive Care Unit, Hospital Universitario Nuestra Señora de Candelaria, Santa Cruz de Tenerife, Spain; 3 Intensive Care Unit, Hospital Universitario Dr. Negrín, Las Palmas de Gran Canaria, Spain; 4 Intensive Care Unit, Hospital Clínico Universitario de Valencia, Valencia, Spain; 5 Intensive Care Unit, Hospital San Jorge de Huesca, Huesca, Spain; 6 Intensive Care Unit, Hospital Insular, Las Palmas de Gran Canaria, Spain; 7 Laboratory Department, Hospital Universitario de Canarias, La Laguna, Santa Cruz de Tenerife, Spain; 8 Atherosclerosis Research Laboratory, CIMA, University of Navarra, Pamplona, Spain; 9 Research Unit, Hospital Universitario de Canarias, La Laguna, Santa Cruz de Tenerife, Spain; University of São Paulo, Brazil

## Abstract

**Objective:**

Higher circulating levels of tissue inhibitor of matrix metalloproteinases (TIMP)-1 at the time of severe sepsis diagnosis have been reported in nonsurviving than in surviving patients. However, the following questions remain unanswered: 1) Does TIMP-1/MMP-9 ratio differ throughout the first week of intensive care between surviving and non-surviving patients? 2) Is there an association between TIMP-1/MMP-9 ratio and sepsis severity and mortality during such period? 3) Could TIMP-1/MMP-9 ratio during the first week be used as an early biomarker of sepsis outcome? 4) Is there an association between TIMP-1/MMP-9 ratio and coagulation state and circulating cytokine levels during the first week of intensive care in these patients? The present study sought to answer these questions.

**Methods:**

Multicenter, observational and prospective study carried out in six Spanish Intensive Care Units (ICUs) of 295 patients with severe sepsis. Were measured circulating levels of TIMP-1, MMP-9, tumour necrosis factor (TNF)-alpha, interleukin (IL)-10 and plasminogen activator inhibitor (PAI)-1 at day 1, 4 and 8. End-point was 30-day mortality.

**Results:**

We found higher TIMP-1/MMP-9 ratio during the first week in non-surviving (n = 98) than in surviving patients (n = 197) (p<0.01). Logistic regression analyses showed that TIMP-1/MMP-9 ratio at days 1, 4 and 8 was associated with mortality. Receiver operating characteristic (ROC) curves showed that TIMP-1/MMP-9 ratio at days 1, 4 and 8 could predict mortality. There was an association between TIMP-1/MMP-9 ratio and TNF-alpha, IL-10, PAI-1 and lactic acid levels, SOFA score and platelet count at days 1, 4 and 8.

**Conclusions:**

The novel findings of our study were that non-surviving septic patients showed persistently higher TIMP-1/MMP-9 ratio than survivors ones during the first week, which was associated with severity, coagulation state, circulating cytokine levels and mortality; thus representing a new biomarker of sepsis outcome.

## Introduction

Sepsis represents a systemic response of the immune system to infection leading to high mortality and costs [1], [2]. Matrix metalloproteinases (MMPs) are a family of zinc-containing endoproteinases that are implicated in degradation and remodelling of the extracellular matrix (ECM) [3]; and also in proteolysis of intracellular protein [4]–[6], and are involved in innate immune defence and apoptosis. The regulation of MMP activity is complex and occurs at several levels [7], such as transcriptional (MMP gene expression in cells) which is modified by TNF-α, interleukin-1β and transforming growth factor (TGF)-β; post- transcriptional (stability of MMP transcripts in cells) which is influenced by glucocorticoids and TGF-β; translational (release of MMP from cells) which is regulated by plasmin and thrombin; and post-translational (activation of MMPs after the release) which is affected by oxidative stress, nitrosative stress, phosphorylation [8], proteolysis and tissue inhibitors of matrix metalloproteinases (TIMPs). MMPs and TIMPs help modulate inflammatory [9] and prothrombotic responses [10], [11].

Previous clinical studies including our own have shown higher circulating levels of MMP-9 and TIMP-1 in septic patients than in controls [12]–[22], and higher levels of TIMP-1 [15], [19], [20], [23] at the time of severe sepsis diagnosis in non-surviving than in surviving patients. In addition, an association between circulating TIMP-1 and plasminogen activator inhibitor (PAI)-1 levels in septic patients at severe sepsis diagnosis has been reported [23]. However, the following questions remain unanswered: 1) Does TIMP-1/MMP-9 ratio differ throughout the first week of intensive care between surviving and non-surviving septic patients? 2) Is there an association between TIMP- 1/MMP-9 ratio and sepsis severity during this period? 3) Is there an association between TIMP-1/MMP-9 ratio during the first week and sepsis mortality? 4) Could TIMP- 1/MMP-9 ratio be used as an early biomarker of sepsis outcome? 5) Is there an association between TIMP-1/MMP-9 ratio and coagulation state during the first week of intensive care in these patients? 6) Is there an association between TIMP-1/MMP-9 ratio and circulating cytokine levels during the first week of intensive care in septic patients? The present study sought to answer these questions. We focus on the determination of the TIMP-1/MMP-9 ratio because in previous studies we assessed serum TIMP-1 and MMP-9 levels [19], [23], and because MMP-9 is inhibited by TIMP-1.

## Methods

### Design and Subjects

A prospective, multicenter observational study was carried out in six Spanish intensive care units (ICUs). The Institutional Ethic Review Boards of the six hospitals approved this study: Hospital Universitario de Canarias (La Laguna. Santa Cruz de Tenerife. Spain), Hospital Universitario Nuestra Señora de Candelaria (Santa Cruz de Tenerife. Spain), Hospital Universitario Dr. Negrín (Las Palmas de Gran Canaria. Spain), Hospital Clínico Universitario de Valencia (Valencia. Spain), Hospital San Jorge (Huesca. Spain) and Hospital Insular (Las Palmas de Gran Canaria. Spain). Written informed consent from the patients or from their family members was obtained.

Patients admitted to the ICU of the participating hospitals and meeting the criteria for severe sepsis were included in the present study. The diagnosis of severe sepsis was established according to the International Sepsis Definitions Conference [24]. Exclusion criteria were: age <18 years, pregnancy, lactation, human immunodeficiency virus (HIV), white blood cell count <103/mm3, solid or hematological tumors or immunosuppressive, steroid or radiation therapy.

### Variables recorded

The following variables were recorded for each patient: sex, age, diabetes mellitus, chronic obstructive pulmonary disease (COPD), ischemic heart disease, site of infection, microorganism responsible, bloodstream infection, antimicrobial treatment, pressure of arterial oxygen/fraction of inspired oxygen (PaO2/FIO2), creatinine, bilirubin, leukocytes, lactic acid, platelets, international normalized ratio (INR), activated partial thromboplastin time (aPTT), Sepsis-related Organ Failure Assessment [SOFA] score [25] and Acute Physiology and Chronic Health Evaluation (APACHE)-II score [26]. We assessed survival at 30 days as the endpoint.

### Proteolytic, inflammatory and prothrombotic marker assays

Blood samples were collected at days 1, 4 and 8 of ICU admission. Serum separator tubes were used to determine MMP-9, TIMP-1, TNF-alpha and IL-10 concentrations, and venous citrated plasma tubes to determine PAI-1 concentration. Venous blood samples were taken and centrifuged within 30 minutes at 1000 g for 15 min, and frozen at −80°C until assayed.

The assay of MMP-9 and TIMP-1 was centralized in Atherosclerosis Research Laboratory of CIMA-University of Navarra (Pamplona, Spain). Specific ELISA (Quantikine, R&D Systems, Abingdon, United Kingdom) was used according to manufacturer's instructions with a serum dilution of 1∶80 and 1∶100 respectively. The interassay coefficients of variation (CV) were <8% (n = 20) and the detection limits for the assays were 0.156 ng/ml and 0.08 ng/ml respectively.

Assays for TNF-alpha, IL-10 and PAI-1 were performed at the Laboratory Department of the Hospital Universitario de Canarias (La Laguna, Santa Cruz de Tenerife, Spain). TNF-alpha and IL-10 were measured in serum by solid-phase chemiluminescent immunometric assays (Immulite, Siemens Healthcare Diagnostics Products, Llanberis, United Kingdom). The interassay CV were <6.5% (n = 20) and <9.9% (n = 40) respectively, and the detection limits for the assays were 1.7 pg/ml and 1 pg/ml respectively. PAI-1 antigen was assayed by specific ELISA (Imubind Plasma PAI-1 ElisaTM, American Diagnostica, Inc, Stanford, CT, USA). This assay detects latent (inactive) and active forms of PAI-1 and PAI-1 complexes. The interassay CV was <5% (n = 20) and the detection limit for the assay was 1 ng/ml.

### Statistical Methods

Continuous variables are reported as medians and interquartile ranges. Categorical variables are reported as frequencies and percentages. Comparisons of continuous variables between groups were carried out using Wilcoxon, Mann-Whitney or Friedman tests. Comparisons between groups for categorical variables were carried out with chi-square test. Logistic regression analyses were applied to determine the independent contribution of TIMP-1/MMP-9 ratio at days 1, 4 and 8 on 30-day mortality, controlling for SOFA score and diabetes mellitus. To avoid the collinearity effect, we included only SOFA score and age as co-predictors. Odds ratio (OR) and 95% confidence intervals (CI) were calculated as measures of the clinical impact of the predictor variables. We plotted receiver operating characteristic (ROC) curves using survival at 30 days as the classification variable and TIMP-1/MMP-9 ratio at days 1, 4 and 8 as prognostic variables. The association between continuous variables was assessed using Spearman's rank correlation coefficient. A P value of less than 0.05 was considered statistically significant. Statistical analyses were performed with SPSS 17.0 (SPSS Inc., Chicago, IL, USA) and NCSS 2000 (Kaysville, Utah).

## Results

Comparison of demographic and clinical parameters between non-surviving (n = 98) and surviving septic patients (n = 197) is shown in Table 1. Non-survivors were older and showed higher diabetes mellitus, creatinine, lactic acid, INR, aPTT, SOFA and APACHE-II, and lower platelet count than survivors. No differences were observed regarding sex, COPD, ischemic heart disease, site of infection, microorganism responsible, bloodstream infection and antimicrobial treatment.

**Table 1:** Baseline clinical and biochemical characteristics of survivor and non-survivor patients.

	Survivors (n = 197)	Non-survivors (n = 98)	P
Male sex– n (%)	65 (23.0)	35 (35.7)	0.70
Age - median years (percentile 25–75)	59 (47–69)	64 (55–74)	0.004
Diabetes Mellitus – n (%)	49 (24.9)	38 (38.8)	0.01
COPD - n (%)	26 (13.2)	13 (13.3)	0.99
Ischemic heart disease - n (%)	21(11.0)	10 (10.3)	0.99
Site of infection			0.67
Respiratory - n (%)	111 (56.3)	58 (59.2)	
Abdominal - n (%)	55 (27.9)	25 (25.5)	
Urinary - n (%)	11 (5.6)	4 (4.1)	
Skin - n (%)	9 (4.6)	4 (4.1)	
Endocarditis - n (%)	6 (3.0)	5 (5.1)	
Arthritis - n (%)	0	1 (1.0)	
CNS- n (%)	5 (2.5)	1 (1.0)	
Microorganism responsibles			
Unknown- n (%)	97 (49.2)	51 (52.0)	0.71
Gram-positive- n (%)	50 (25.4)	25 (25.5)	0.99
Gram-negative- n (%)	50 (25.4)	21 (21.4)	0.47
Fungii- n (%)	4 (2.0)	4 (4.1)	0.45
Anaerobe - n (%)	2 (1.0)	1 (1.0)	0.99
Bloodstream infection - n (%)	30 (15.2)	17 (17.3)	0.74
Empiric antimicrobial treatment adequate			0.68
Unknown due to negative cultures - n (%)	97 (49.2)	52 (53.1)	
Unknown due to diagnosis by antigenuria - n (%)	14 (7.1)	4 (4.1)	
Adequate - n (%)	82 (41.6)	39 (39.8)	
Inadequate - n (%)	4 (2.0)	3 (3.1)	
Treatment with betalactamic more aminoglycoside - n (%)	43 (22.1)	24 (24.5)	0.66
Treatment with betalactamic more quinolone - n (%)	101 (51.8)	51 (52.0)	0.99
Pa0_2_/FI0_2_ ratio - median (percentile 25–75)	180 (122–270)	168 (101–240)	0.12
Creatinine (mg/dl) - median (percentile 25–75)	1.30 (0.84–2.10)	1.60 (1.00–2.90)	0.009
Bilirubin (mg/dl) - median (percentile 25–75)	0.90 (0.50–1.47)	0.90 (0.50–2.30)	0.41
Leukocytes - median*10^3^/mm^3^ (percentile 25–75)	14.7 (9.2–20.0)	15.0 (7.1–20.6)	0.85
Lactic acid - median mmol/L (percentile 25–75)	2.00 (1.10–3.40)	3.55 (1.60–6.00)	<0.001
Platelets - median*10^3^/mm^3^ (percentile 25–75)	197 (131–273)	132 (63–222)	<0.001
INR - median (percentile 25–75)	1.27 (1.10–1.51)	1.42 (1.14–1.89)	0.008
aPTT - median seconds (percentile 25–75)	32 (28–39)	36 (29–45)	0.008
SOFA score - median (percentile 25–75)	9 (7–11)	11 (9–14)	<0.001
APACHE-II score - median (percentile 25–75)	19 (15–23)	24 (19–29)	<0.001
TIMP-1– median ng/ml (percentile 25–75)	548 (372–732)	750 (479–1020)	<0.001
MMP-9 - median ng/ml (percentile 25–75)	760 (359–1208)	533 (231–952)	0.003
TIMP-1/MMP-9 ratio (percentile 25–75)	0.71 (0.40–1.54)	1.26 (0.64–3.86)	0.003
TNF-alpha - median pg/ml (percentile 25–75)	30 (20–50)	36 (18–74)	0.24
IL-10 - median pg/ml (percentile 25–75)	11 (6–37)	38 (9–119)	<0.001
PAI-1 - median ng/ml (percentile 25–75)	36 (21–62)	58 (33–84)	<0.001
			

COPD  =  chronic obstructive pulmonary disease; CNS  =  Central Nervous System; PaO_2_/FIO_2_  =  pressure of arterial oxygen/fraction inspired oxygen; INR  =  international normalized ratio; aPTT  =  activated partial thromboplastin time; SOFA  =  Sepsis-related Organ Failure Assessment score; APACHE II  =  Acute Physiology and Chronic Health Evaluation; MMP  =  matrix metalloproteinase; TIMP  =  tissue inhibitor of matrix metalloproteinase; TNF  =  tumour necrosis factor; IL  =  Interleukin; PAI  =  Plasminogen activator inhibitor.

**Figure 1:**
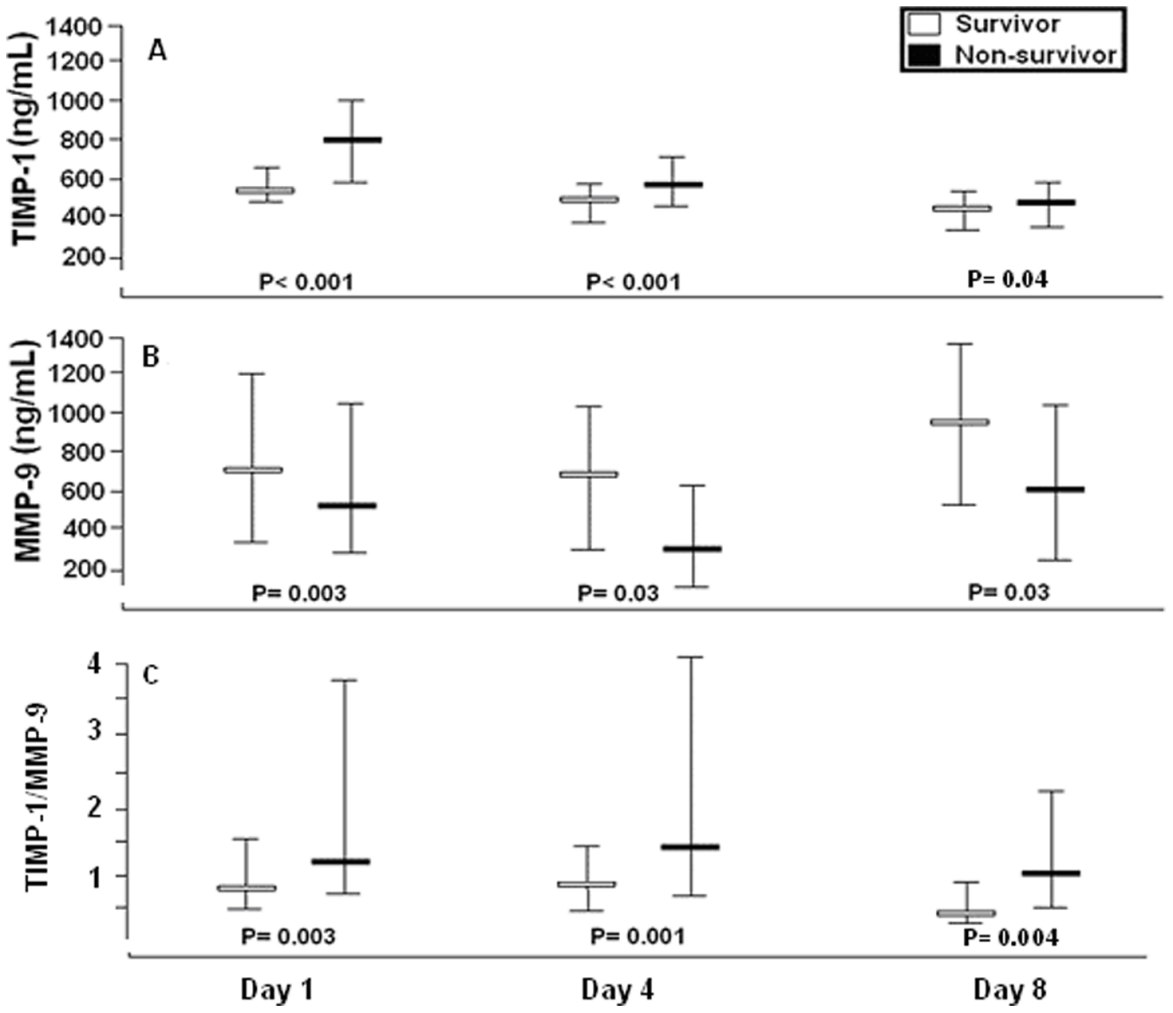
Serum MMP-9, TIMP-1 and TIMP-1/MMP-9 ratio in survivor and non-survivor septic patient showed as median (percentiles 25–75). MMP  =  Matrix metalloproteinase; TIMP  =  Tissue inhibitor of matrix metalloproteinase.

**Figure 2:**
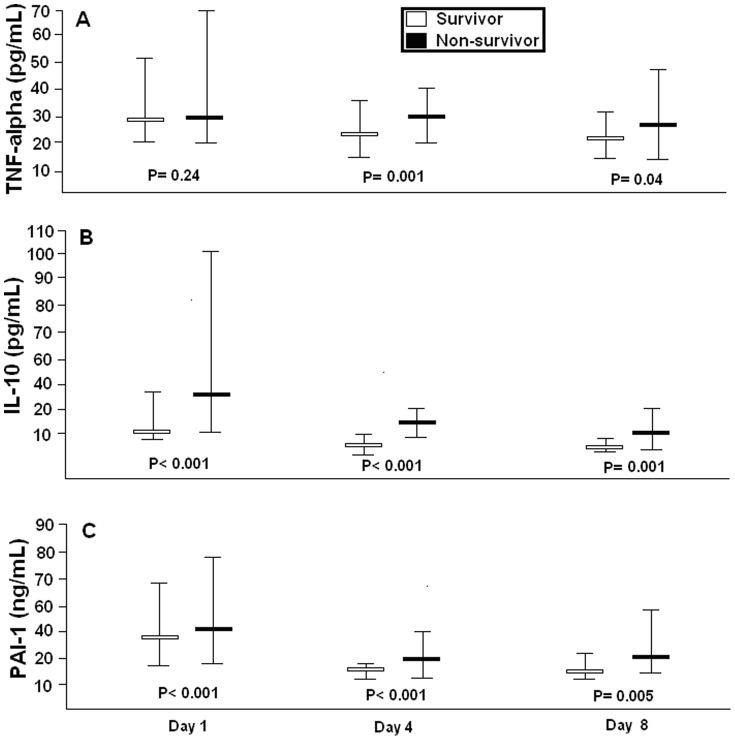
Serum TNF-alpha and IL-10 levels and plasma PAI-1 levels in survivor and non-survivor septic patients showed as median (percentile 25–75). TNF  =  tumour necrosis factor; IL  =  Interleukin; PAI  =  Plasminogen activator inhibitor.

Non-surviving septic patients showed higher serum TIMP-1 levels, lower serum MMP-9 levels and higher TIMP-1/MMP-9 ratio than survivors at days 1, 4 and 8 (Table 1, Figure 1). Additionally, non-surviving septic patients exhibited higher PAI-1 and IL- 10 levels at days 1, 4 and 8, and higher TNF-alpha at day 4 and 8 than survivors (Table 1 and Figure 2).

Logistic regression analysis showed that TIMP-1/MMP-9 ratios at day 1 (OR = 1.11; 95% CI = 1.011–1.223; p = 0.03), day 4 (OR = 1.27; 95% CI = 1.004–1.617; p = 0.047) and day 8 (OR = 1.76; 95% CI = 1.041–2.977; p = 0.03) were associated with higher mortality at 30 days, controlling for SOFA score (Table 2).

**Table 2:** Logistic regression analyses to predict 30-day mortality.

		Odds Ratio		95% Confidence IntervalP	
**First model**:					
TIMP-1/MMP-9 ratio at day 1		1.11	1.011–1.223		0.03
SOFA score at day 1		1.17	1.083–1.268		<0.001
Diabetes mellitus		1.99	1.137–3.489		0.02
**Second model**:					
TIMP-1/MMP-9 ratio at day 4		1.27	1.004–1.617		0.047
SOFA score at day 4		1.15	1.050–1.267		0.003
Diabetes mellitus		2.76	1.182–6.425		0.02
**Third model**:					
TIMP-1/MMP-9 ratio at day 8		1.76	1.041–2.977		0.03
SOFA score at day 8		1.19	1.064–1.334		0.002
Diabetes mellitus		1.93	0.652–5.721		0.23

TIMP  =  tissue inhibitor of matrix metalloproteinase; MMP  =  matrix metalloproteinase; SOFA  =  Sepsis-related Organ Failure Assessment score.

**Figure 3:**
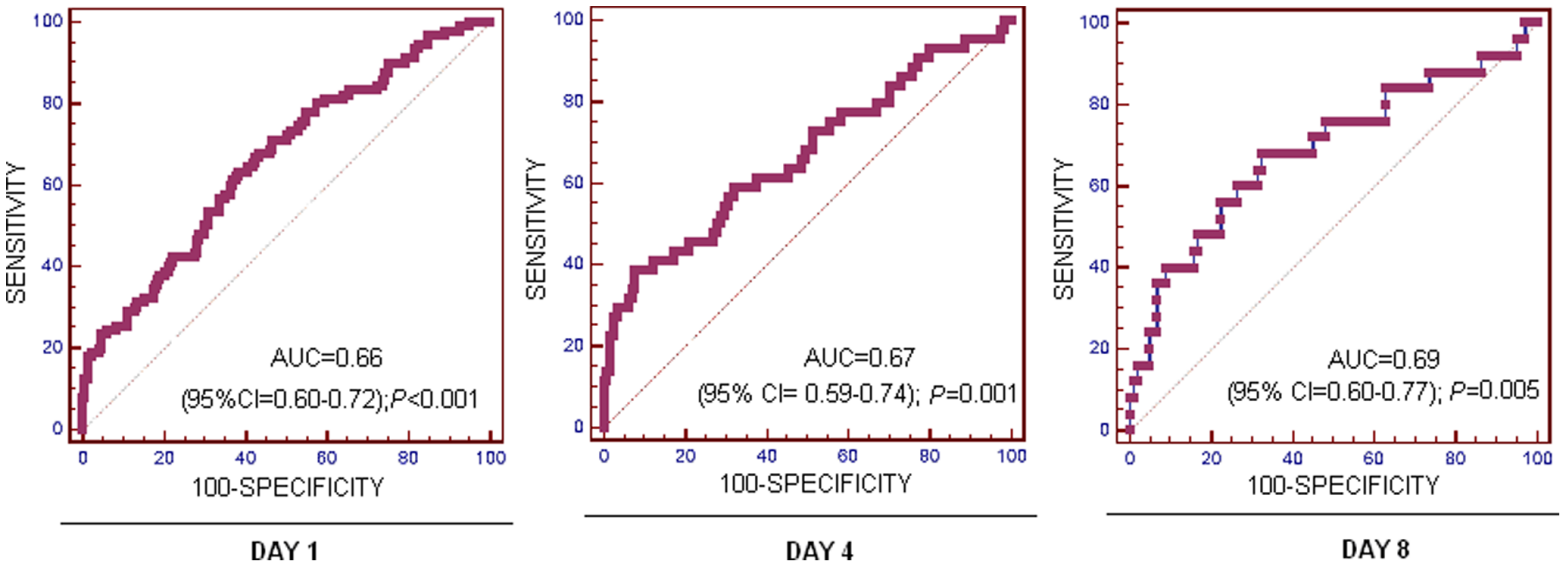
Receiver operation characteristic analyses using TIMP-1/MMP-9 ratio at day 1, 4 and 8 after admission as predictor of mortality at 30 days in severe septic patients.

On ROC curve analysis, the area under the curve of TIMP-1/MMP-9 ratios at day 1, 4 and 8 to predict 30-day survival were 0.66 (95% CI = 0.60–0.72; p<0.001), 0.67 (95% CI = 0.59–0.74; p = 0.001) and 0.69 (95% CI = 0.60–0.77; p = 0.005) respectively, (Figure 3).

As shown in Table 3, TIMP-1/MMP-9 ratios at days 1, 4 and 8 correlated with circulating TNF-alpha, IL-10, PAI-1 and lactic acid levels, SOFA score, and platelet count, and at days 1 and 4 with INR and aPTT.

**Table 3:** Correlations between circulating TIMP-1/MMP-9 ratio levels and TNF-alpha, IL-10, PAI-1, lactic acid, SOFA score, platelet, INR and aPTT at days 1, 4 and 8.

	Day 1	Day 4	Day 8
TNF-alpha	rho = 0.45; p<0.001	rho = 0.20; p = 0.04	rho = 0.25; p = 0.01
IL-10	rho = 0.51; p<0.001	rho = 0.33; p<0.001	rho = 0.19; p = 0.05
PAI-1	rho = 0.36; p<0.001	rho = 0.34; p<0.001	rho = 0.23; p = 0.01
Lactic acid	rho = 0.45; p<0.001	rho = 0.31; p<0.001	rho = 0.21; p = 0.02
SOFA score	rho = 0.51; p<0.001	rho = 0.31; p<0.001	rho = 0.36; p<0.001
Platelet	rho = −0.46; p<0.001	rho = −0.34; p<0.001	rho = −0.26; p = 0.004
INR	rho = 0.49; p<0.001	rho = 0.23; p = 0.01	rho = 0.11; p = 0.26
aPTT	rho = 0.34; p<0.001	rho = 0.22; p = 0.01	rho = 0.08; p = 0.41
Age	rho = 0.12; p = 0.02	rho = 0.19; p = 0.01	rho = 0.25; p = 0.003

TIMP  =  tissue inhibitor of matrix metalloproteinase; MMP  =  matrix metalloproteinase; TNF  =  tumour necrosis factor; IL  =  Interleukin; PAI  =  plasminogen activator inhibitor; SOFA  =  Sepsis-related Organ Failure Assessment score; INR  =  international normalized ratio; aPTT  =  activated partial thromboplastin time.

## Discussion

A novel finding of our study was that non-surviving septic patients showed persistently higher circulating levels TIMP-1 levels, lower circulating MMP-9 levels and higher TIMP-1/MMP-9 ratio than survivors during the first week of ICU stay. In addition, we found for the first time that the TIMP-1/MMP-9 ratio during the first week was associated with mortality according to the results of logistic regression analyses. Two previous studies exploring MMP levels during the course of sepsis found no significant differences between surviving and non-surviving patients regarding TIMP-1 [21] and MMP-9 levels [21], [22]. The higher sample size of our study (295 patients) compared with the smaller size (20 and 44 patients respectively) of previous studies [21], [22] could explain the significant differences reported between surviving and non- surviving patients.

In a previous study by our team we found that TIMP-1 levels at the time of severe sepsis diagnosis could be used as a biomarker of sepsis outcome [19]. A novel finding of the current study was that TIMP-1/MMP-9 ratio during the first week could be used as a biomarker of sepsis outcome according to the results of ROC curve analysis. In addition, we previously reported that circulating TIMP-1 levels were associated with TNF-alpha, IL-10, PAI-1, SOFA score, lactic acid levels, platelet count, aPTT and INR [19]. Another novel finding of the present study was a significant association between TIMP-1/MMP-9 ratio and circulating cytokine levels, coagulation parameters and SOFA score during the first week.

Taken together, these results indicate that TIMP-1 and MMP-9 levels could play a pathophysiological role in septic patients. MMP-9 inhibits platelet aggregation [27], [28] and an association between circulating TIMP-1 and PAI-1 levels has been found [29], [30]. In addition, an association between PAI-1 levels and sepsis mortality has been reported [31]–[33]. Thus, it may be that the higher circulating levels TIMP-1 levels, lower circulating MMP-9 levels and higher TIMP-1/MMP-9 ratio found in non-surviving patients during the first week could favour a prothrombotic state and contribute to capillary thrombosis, multiple organ dysfunction, and death in septic patients.

From a therapeutic perspective, the development of modulators of MMP/TIMP activity could be used as a new class of drugs for the treatment of severe sepsis [34], [35].

The strengths of our study were the large sample size and the follow-up of circulating TIMP-1, MMP-9, TNF-alpha, IL-10, and PAI-1 levels throughout the first week after diagnosis of severe sepsis. However, our study has certain limitations. First, we did not analyze other MMPs and TIMPs; and it could be interesting to describe levels of other MMPs and TIMPs, and the ratios between them. Second, measurement of other cytokines and coagulation markers would be desirable in order to better evaluate the relationship between proteolysis, inflammation and coagulation in these patients. Third, we analyzed serum levels of MMP-9 and TIMP-1 but plasma determination is preferable because there could be a release of several MMPs or TIMPs from platelets in serum [36]–[39].

In conclusion, the novel findings of our study were that non-surviving septic patients showed persistently higher TIMP-1/MMP-9 ratio than survivors during the first week, which was associated with severity, coagulation state, circulating cytokine levels and mortality, thus representing a new biomarker of sepsis outcome.
